# Perspective on Schistosomiasis Drug Discovery: Highlights from a Schistosomiasis Drug Discovery Workshop at Wellcome Collection, London, September 2022

**DOI:** 10.1021/acsinfecdis.3c00081

**Published:** 2023-04-21

**Authors:** Nicola Caldwell, Rana Afshar, Beatriz Baragaña, Amaya L. Bustinduy, Conor R. Caffrey, James J. Collins, Daniela Fusco, Amadou Garba, Mark Gardner, Mireille Gomes, Karl F. Hoffmann, Michael Hsieh, Nathan C. Lo, Case W. McNamara, Justin Komguep Nono, Gilda Padalino, Kevin D. Read, Meta Roestenberg, Thomas Spangenberg, Sabine Specht, Ian H. Gilbert

**Affiliations:** † Wellcome Centre for Anti-Infectives Research, Drug Discovery Unit, Division of Biological Chemistry and Drug Discovery, University of Dundee, Dundee DD1 5EH, United Kingdom; ‡ Global Health Institute of Merck, a subsidiary of Merck KGaA, Darmstadt, Germany, Ares Trading S.A., Route de Crassier 1, 1262 Eysins, Switzerland; § Department of Clinical Research, London School of Hygiene & Tropical Medicine, Keppel Street, London WC1E 7HT, United Kingdom; ∥ Center for Discovery and Innovation in Parasitic Diseases, Skaggs School of Pharmacy and Pharmaceutical Sciences, University of California San Diego, 9500 Gilman Drive, MC0657, La Jolla, California 92093, United States; ⊥ Department of Pharmacology, UT Southwestern Medical Center, Forest Park Road, Dallas, Texas 75235, United States; # Department of Infectious Disease Epidemiology, Bernhard Nocht Institute of Tropical Medicine, 20359 Hamburg, Germany; ¶ German Center for Infection Research (DZIF), Hamburg-Borstel-Lübeck-Riems, 38124 Brunswick, Germany; ⊗ Department of Control of Neglected Tropical Diseases, World Health Organization, 1202 Geneva, Switzerland; ∇ Salvensis Ltd., 27 New Dover Rd., Canterbury, Kent CT1 3DN, United Kingdom; × Department of Life Sciences (DLS), Aberystwyth University, Edward Llwyd Building, Aberystwyth SY23 3DA, United Kingdom; ☆ Division of Urology, Children’s National Hospital, and Department of Urology, George Washington University, Washington, D.C. 20010, United States; □ Division of HIV, Infectious Diseases, and Global Medicine, University of California San Francisco, San Francisco, California 94110, United States; ○ Calibr, a division of Scripps Research, North Torrey Pines Road, La Jolla, California 92037, United States; △ Unit of Immunobiology and Helminth Infections, Institute of Medical Research and Medicinal Plant Studies (IMPM), Ministry of Scientific Research and Innovation, Yaoundé 13033, Cameroon; ▼ School of Pharmacy and Pharmaceutical Sciences, Cardiff University, Redwood Building, King Edward VII Avenue, Cardiff CF10 3NB, United Kingdom; ● Department of Parasitology and Department of Infectious Diseases, Leiden University Medical Centre, 2333 ZA Leiden, The Netherlands; ▲ Drugs for Neglected Diseases Initiative, 1202 Geneva, Switzerland

**Keywords:** schistosomiasis, *Schistosoma*, neglected tropical disease, infectious disease, drug discovery, therapeutics, anthelmintics, target product profile

## Abstract

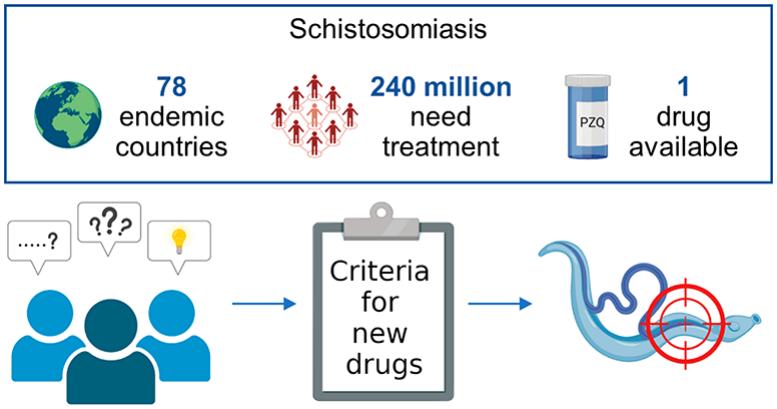

In September 2022, the Drug Discovery Unit at the University of Dundee, UK, organised an international meeting at the Wellcome Collection in London to explore the current clinical situation and challenges associated with treating schistosomiasis. The aim of this meeting was to discuss the need for new treatments in view of the clinical situation and to ascertain what the key requirements would be for any potential new anti-schistosomals. This information will be essential to inform ongoing drug discovery efforts for schistosomiasis. We also discussed the potential drug discovery pathway and associated criteria for progressing compounds to the clinic. To date, praziquantel (PZQ) is the only drug available to treat all species causing schistosomiasis, but it is often unable to completely clear parasites from an infected patient, partially due to its inactivity against juvenile worms. PZQ-mediated mass drug administration campaigns conducted in endemic areas (e.g., sub-Saharan Africa, where schistosomiasis is primarily prevalent) have contributed to reducing the burden of disease but will not eliminate the disease as a public health problem. The potential for *Schistosoma* to develop resistance towards PZQ, as the sole treatment available, could become a concern. Consequently, new anthelmintic medications are urgently needed, and this Perspective aims to capture some of the learnings from our discussions on the key criteria for new treatments.

## Introduction

### Schistosomiasis

Schistosomiasis is an infectious disease caused by parasitic flatworms of the genus *Schistosoma*. After malaria, schistosomiasis is the second most pathogenic human parasitic infection, disproportionately affecting people who live in Low to Middle Income Countries (LMICs).^1^ As a result, it has been targeted for elimination by 2030 in the World Health Organisation (WHO)’s neglected tropical diseases (NTDs) road map entitled “Ending the neglect to attain the sustainable development goals”.^2^ The development of new tools for disease prevention, diagnosis, and treatment is the key to this ambitious agenda.

Three main *Schistosoma* species are responsible for the majority of human infections—*Schistosoma mansoni*, *Schistosoma haematobium*, and *Schistosoma japonicum*. Each species has a different geographic distribution. Both *S. mansoni* and *S. haematobium* are responsible for causing infection in sub-Saharan Africa, which has the highest global prevalence of schistosomiasis. Elsewhere, *S. mansoni* is endemic in areas of South America, including Brazil, Suriname, and Venezuela, and *S. haematobium* is endemic in the Middle East. *S. japonicum* is mainly distributed in China, Indonesia, and the Philippines. There are other minor, less prevalent species that can also cause human disease, including *Schistosoma mekongi* (only found in southern Cambodia and along the Mekong River in Laos), *Schistosoma guineensis*, and *Schistosoma intercalatum* (both found in West and Central Africa).^3^ Hybridised schistosomes, genetic variants resulting from cross-specific worm couplings, have been recently described which display molecular and phenotypic features of both human (*S. haematobium*) and animal (*Schistosoma bovis*, *Schistosoma mattheei*, or *Schistosoma curassoni*) pathogens.^4^ The prevalence of human infection caused by these hybrids is currently unknown, but is of concern.^5^

*Schistosoma* parasites have a complex life cycle. Following asexual replication in intermediate freshwater snail hosts, cercariae are released into water sources and can infect humans through penetration of the skin. Upon entering the circulation, larval schistosomula develop first into juveniles, then into dimorphic adult worms. Adults subsequently migrate to the mesenteric venous system (*S. mansoni* and *S. japonicum*) or the venous plexus of the bladder and other pelvic organs (*S. haematobium*), where they can reside for years as sexually mature male/female pairs. During this time, paired females lay eggs, some of which are excreted in urine or faeces. Eggs which are released into freshwater sources can complete the parasite life cycle, leading to transmission of infection. Eggs which are not excreted can become trapped in the visceral organs, e.g. intestine and liver, which triggers an immune-inflammatory response. The resulting granulomas and fibrosis are responsible for the development of clinical symptoms. Chronic infection can result in severe long-term health consequences such as liver fibrosis, bladder cancer, and urogenital disease.^6^

### Praziquantel

Praziquantel (PZQ), a racemic mixture of biologically active *R* and less active *S* enantiomers,^7^ was approved as an anthelmintic in 1980 and remains the only drug available to treat schistosomiasis. It is widely used due to its low cost and good safety record. However, its rather bitter taste (primarily due to the *S* enantiomer) and large tablet size (partially due to the racemic formulation) have caused issues during the treatment of children. The Pediatric Praziquantel Consortium (www.pediatricpraziquantelconsortium.org) is addressing these limitations by developing a pediatric treatment which consists of only the most active *R* enantiomer and is more appropriately formulated for small children.^8^ Preliminary data from phase 3 clinical trials presented during this meeting showed good efficacy and safety, and improved palatability compared to the current formulation of PZQ. This new PZQ pediatric formulation will be provided as an orally dispersible tablet, with the aim for manufacture by partners in Africa.

Administration of PZQ causes rapid contraction and damage to the tegument (surface) of adult worms. PZQ has been shown to activate a transient receptor potential melastatin ion channel (*Sm*TRPM_PZQ_) causing an influx of calcium and worm paralysis, which leads to a reduction in egg production, and death.^9^

Limited human pharmacokinetic (PK) data in healthy volunteers indicate that PZQ suffers from extensive first pass metabolism and, as a result, systemic exposure in the bloodstream is low.^10^ In the case of *S. mansoni*, which is localised in the mesenteric vein, adult worms encounter PZQ before it is metabolised in the liver. Therefore, PZQ is efficacious despite its poor oral bioavailability. However, exposure to PZQ is lower for *S. haematobium* or juvenile forms of *S. mansoni* due to the different locations of the parasites within the body. Studies with PZQ in an *S. mansoni* mouse model in the presence of cytochrome p450 (CYP) inhibitor 1-aminobenzotriazole (ABT) to increase systemic concentrations of the drug do not show a dose-dependent increase in worm burden reduction. Conversely, co-dosing with a CYP inducer, dexamethasone (DEX), to decrease systemic concentrations of PZQ does not reduce efficacy. These results indicate that high concentrations of PZQ in the mesenteric vein are required to clear adult *S. mansoni* worms and efficacy does not correlate with systemic exposure.^11,12^

Juvenile worms in mouse models of disease do not respond to PZQ treatment and go on to (re)establish infection with the consequent morbidity.^13^ In PZQ-treated individuals harbouring this developmental stage, repeat treatment is often required at 6–8 weeks. Furthermore, exposing parasites to potentially sub-optimal dosing has raised concerns regarding the encouragement of drug resistance.^14,15^ With limited efficacy against juvenile worms and increasing concerns about developing resistance, there is a clear need for alternative treatments that can advance the goal of disease elimination as outlined by WHO.

### Workshop

To understand the current clinical situation and challenges associated with treating schistosomiasis, the Drug Discovery Unit (University of Dundee, UK) organised an international workshop at the Wellcome Collection, London, in September 2022 (see Supporting Information for the program). The aim of this meeting was to bring together researchers working on schistosomiasis around the world and discuss the key requirements for new treatments that may be required in the event of emerging resistance towards PZQ. The meeting was attended by over 50 people and included those working in drug discovery, academics, clinicians, and public health experts. We were particularly keen to capture the views of people who live and work in endemic areas, as a clear understanding of the clinical situation will be essential to inform drug discovery efforts for schistosomiasis. This Perspective highlights the learnings from our discussions and suggestions of key criteria for new anti-schistosomals.

## Clinical Situation

### Schistosomiasis

Schistosomiasis is endemic in 78 countries, and nearly 800 million people worldwide are at risk of infection, with more than 90% of those at risk living in Africa.^16^ As of August 2019, WHO estimated that around 240 million people were in need of preventative chemotherapy with PZQ.^17^

### Mass Drug Administration

In partnership with WHO, Merck KGaA, Darmstadt, Germany, provides up to 250 million tablets per year through their PZQ program. Treatment with PZQ is very cost-effective, with an estimated cost of around $0.10 USD per treatment plus implementation costs. Following WHO guidelines, large-scale treatment with PZQ has been crucial in achieving a reduction in disease prevalence of 60% in sub-Saharan Africa.^18^ However, there have been some limitations in treatment coverage and efficiency in sustainably controlling schistosomiasis. PZQ alone has not been completely successful in eliminating schistosomiasis in mass drug administration (MDA) programs. This is likely due to several factors, including inability to clear juvenile worms, poor PK properties, and possible geographical variability in the responsiveness of the parasite to the standard dosing regimen. Therefore, infection can be persistent. Reduced patient compliance may also be a contributing factor towards persistence of infection. Poor palatability and an increase in adverse events including abdominal pain and dizziness have been noted, which may affect adherence to treatment.

### Female and Male Genital Schistosomiasis

Urogenital schistosomiasis can occur when *S. haematobium* eggs recruit host immune cells to form granulomas which can become trapped in genital tissues and lead to fibrosis. In the case of female genital schistosomiasis (FGS), this presents with characteristic lesions (sandy patches) on the cervix or vaginal wall. Under the current treatment regimen, a single dose of PZQ is insufficient to resolve these lesions. Merck KGaA, Darmstadt, Germany, is the primary funder of a study to examine whether repeat dosing with PZQ has a positive effect on the lesions compared to the current single-dose treatment. As well as the morbidity due to the granulomas and fibrosis, FGS is thought to increase the likelihood of the patient contracting sexually transmitted infections (STIs), including HIV.

The Fusco group at the Bernhard Nocht Institute for Tropical Medicine are carrying out studies on FGS in Madagascar, which has a high prevalence of schistosomiasis. It is estimated that around 52.1% of the total population are infected with either *S. mansoni* or *S. haematobium*.^19^ Treatment of FGS is complicated by a lack of data on the chronic manifestation of the disease and the number of sufferers. Furthermore, the public health system in Madagascar is fragile due to territorial, cultural, and political reasons, so many women do not access regular health care. As the symptoms of FGS are linked to STIs, there is also stigma associated with seeking medical care. Therefore, in order to generate demand for treatment, it was important to first raise awareness of the prevalence of the disease in endemic areas. The FIRM-UP study (**F**emale Genital Schistosomiasis **i**n **r**ural **M**adagascar: improving community **u**nderstanding and **p**romoting integration into primary health care services) aimed to create awareness of FGS at a community level through posters, TV, and radio communications, and carry out a screening campaign to identify the impact of FGS on the population.^20^ Due to a lack of adequate diagnostic tools, FGS was diagnosed through visual inspection using colposcopy, and suspected cases were followed up with treatment by PZQ. From a sample size of 434 women, almost 90% tested positive for FGS indicating a very high, previously undiagnosed, prevalence of infection. This targeted campaign to diagnose and raise awareness for FGS was critical to identify patients who needed to be treated.

Male genital schistosomiasis (MGS) is also thought to be highly prevalent, but, similarly to FGS, it is poorly characterised and there is a lack of data available to fully understand the extent of the disease. MGS commonly presents with genital disfigurement, hematospermia, hematuria, and genital pain—symptoms which are often misattributed to STIs. Chronic infection may be associated with infertility and sexual dysfunction. However, diagnosis can be difficult as transabdominal ultrasound studies focussed on bladder pathology can overlook internal infection in the male genital tract. Moreover, current methods of counting the number of eggs in ejaculate can be insensitive and do not correlate with symptoms exhibited by patients. Again, PZQ is the primary treatment, but the current dosing regimen as outlined by MDA guidelines is not curative for MGS. Further studies are needed to assess whether changing dosage or dosing regimens of PZQ would offer improved outcomes.

### Need for New Therapeutic Options

Although PZQ treatment has been successful in controlling disease morbidity and prevalence, its lack of activity against juvenile parasites means that repeat dosing is frequently necessary. As a result, it is unlikely that PZQ alone will achieve elimination goals with single-dose treatments as recommended by MDA guidelines.^17^ Whilst killing adult worms is beneficial at reducing the number of eggs released in faeces and urine, and hence the overall intensity of disease, resolution of clinical symptoms often takes years to accomplish. It was agreed that a drug which targeted eggs directly or disrupted granulomas would carry the risk of a widespread antigen release into the tissues and circulation. However, activity against juvenile stages of the parasite in addition to adult worms could limit egg production and prevent granuloma formation before fibrosis becomes established. Overall, a new treatment that addresses the limitations of PZQ would be beneficial for use in clinical settings.

In addition to exploring new treatments, there is a clear need to develop more accurate diagnostic tools, particularly for urogenital schistosomiasis. Current methods such as using colposcopy to diagnose FGS cannot be easily applied in endemic settings. There are also socioeconomic barriers to accessing treatment for FGS and MGS and the use of targeted campaigns to raise awareness of the disease would be important to consider when trying to integrate new treatment regimens within public health systems.

## Suggested Criteria for New Treatments

### Uses for New Treatments

Following presentations that highlighted the current clinical situation, discussion sessions were held to examine the key requirements for new treatments. Suggested criteria that were proposed for the treatment of uncomplicated schistosomiasis infection are shown in Table 1 along with some outstanding questions that need to be addressed. These are questions that will require further input from the community to define suitable criteria for new treatments.

**Table 1:**
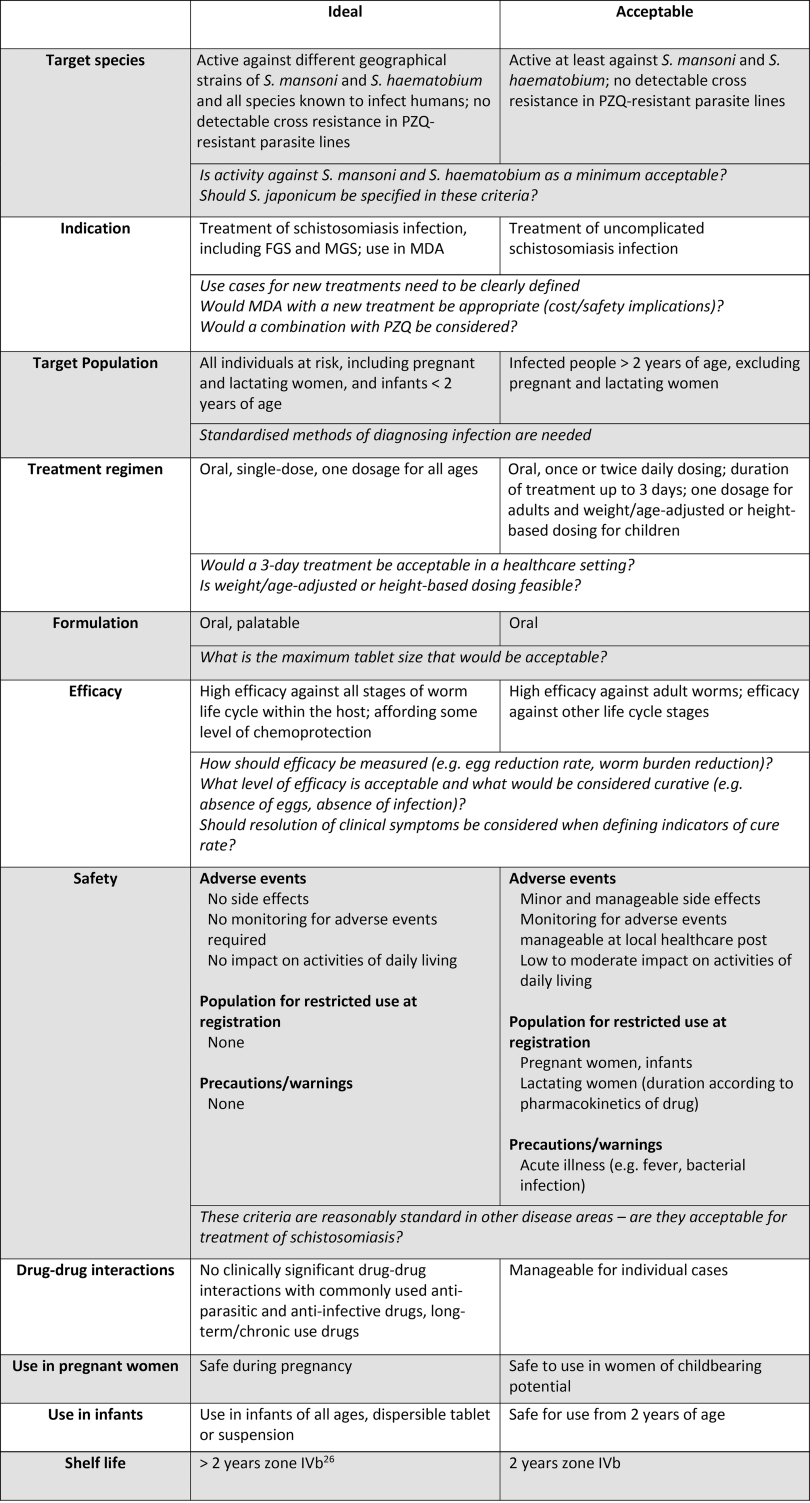
Suggestions for Ideal and Acceptable Criteria for Treating Uncomplicated Schistosomiasis and Outstanding Questions That Need to Be Addressed

As outlined above, a major limitation of PZQ is its inactivity against juvenile worms, which often necessitates repeat dosing within MDA programs in endemic areas. It was agreed that any new treatment should address this issue and ideally be active against schistosomula, juvenile, and adult stages of parasite development in humans. Since *S. mansoni* and *S. haematobium* represent 92% of the global disease burden, targeting at least these species would be acceptable as a minimum.

A new treatment could be used in two different settings: MDA or a test and treat clinical application. Whilst the use of a new drug in a combination therapy with PZQ could be feasible, the cost of development would significantly increase which would limit its use in MDA programs. It was agreed that the cost-effectiveness of any new treatment should be considered throughout development, but it is not appropriate to benchmark against the cost of PZQ which is largely provided through global donation programs.

For a test and treat clinical application, it was discussed that a treatment regimen of up to 3 days would likely be acceptable within a healthcare setting. However, the ideal scenario would be a single-dose treatment, as there are likely to be compliance issues with multiple doses. As reduced compliance with PZQ treatment is often attributed to its bitter taste, palatability is also an important consideration.

### Determining Efficacy

A key point discussed was how efficacy of a new drug should be determined and what level of efficacy would be deemed to be acceptable. Common methods of diagnosing schistosomiasis by counting eggs in faeces or urine are labour intensive, and procedures are not standardised in the field so efficacy can be difficult to determine. The number of eggs produced are also thought to vary during the day, and egg reduction rates are not a good indicator of cure rate as evidenced by the reestablishment of egg-induced morbidity after initial PZQ treatment. Measuring levels of worm-specific antigens in urine or blood, or qPCR detection of parasite DNA in blood, urine, or faeces may be more sensitive for diagnosing infection prior to egg laying but there is limited data available on the correlation with levels of infection. Circulating anodic antigen (CAA) and circulating cathodic antigen (CCA) are released by adult worms into the host bloodstream via regurgitation of undigested gut contents and then excreted in urine. Therefore, they can be used as a biomarker for schistosomiasis infection.^21^

A recent clinical study indicates that diagnostics based on egg counting tend to over-estimate the cure rate of PZQ. More sensitive methods, that measure worm-derived antigens, indicate that parasites can remain in humans even after multiple treatments with PZQ.^22^ As worms can still be detected in the absence of eggs, this suggests that infection is persistent and previously stated cure rates of 70–80% for PZQ may be an over-estimate, giving further impetus for the development of new treatments.^23,24^

It was discussed that resolution of clinical symptoms should also be taken into consideration when defining a set of indicators for cure rate as symptomatic treatment may be required, particularly in the case of urogenital schistosomiasis. It is possible that anti-inflammatories, if given following treatment with an anti-schistosomal drug, could have some therapeutic utility in managing the ongoing symptoms of FGS/MGS. Improving fertility could also be considered as an indicator of an efficacious treatment for urogenital schistosomiasis.

Identifying a suitable diagnostic tool for use in the field and standardising methods of determining efficacy are essential. Currently there are no diagnostic tools for FGS and MGS that could be implemented widely in the field. However, the WHO has recently established a technical working group to outline diagnostic Target Product Profiles (TPPs) for FGS, similarly to those they have previously published for schistosomiasis control programs.^25,26^

## Desirable Compound Profiles

The definition of desirable compound properties for a preclinical drug candidate for schistosomiasis is important to guide drug discovery in this area. There were discussions about which aspects would be critical to understand when developing new anti-schistosomals.

### Targeting All Developmental Stages

A modelling study carried out at the University of California San Francisco, presented during this meeting, suggests that killing adult worms has the greatest impact on reducing parasite burden. There will be additional benefit from an anthelmintic drug that kills schistosomula and juvenile worms, but the magnitude of this benefit may depend on the epidemiologic environment and public health goals. However, as described above, activity against schistosomula, juveniles, and adult worms is important for preventing the development of chronic schistosomiasis caused by immune responses towards eggs laid by sexually mature adult worms.

Therefore, while it is agreed that adult worms residing in the mesenteric veins and vesicular/pelvic venous plexus need to be killed by a new anti-schistosomal drug, schistosomula and juvenile worms present in other tissues (e.g., skin and lung) and blood should additionally be targeted for elimination. To achieve this, a compound with good tissue penetration is likely to be required to reach all areas in the body in which the parasite resides. A compound with a long half-life would also have the benefit of providing a limited amount of chemoprotection against reinfection which should help in reducing transmission and aid the goal of disease elimination. However, a compound with a long half-life and substantial tissue distribution would increase the risk of toxicity and drug–drug interactions which would be of concern in cases where patients require other drugs to treat additional diseases or co-infections.

Although activity against all human-infective life cycle stages of the parasite is desirable, it has been observed during *ex vivo* screening of compound libraries to identify new anti-schistosomal compounds that activity against schistosomula does not always correlate with activity against adult worms. It is not clear if this is due to different biology, for example if a biological pathway is more important in one life cycle stage compared to another, or if there are varying levels of target expression in different life cycle stages.^27^ It could be due to other factors such as different levels of compound uptake by adult worms compared to schistosomula. Aberystwyth University and the University of Dundee are developing a compound accumulation assay to better understand the degree to which compounds are accumulating within the worms and how this correlates with properties such as permeability, efflux, and metabolism.

### Cidality

The question was raised about whether a cidal compound (one which kills the parasite) would be needed or if a static compound (one which inhibits parasite growth) would be sufficient. It was agreed that cidality would almost certainly be required in order to remove all of the parasites.

### Targeting Eggs

It was agreed that designing active compounds against schistosomula, juvenile, and adult forms of the parasite was desirable but there was considerable discussion around whether it would also be advantageous for a compound to clear the eggs. As discussed above, the presence of the eggs is responsible for driving pathogenesis during the chronic stages of infection due to their ability to induce tissue granulomas and fibrosis. However, a concern was raised that a drug which rapidly killed eggs could trigger an immediate and potentially dangerous immune response due to the simultaneous release of antigens from thousands of tissue-trapped eggs. To limit the risk of inducing immune responses upon killing thousands of eggs simultaneously, it was suggested that the best way to tackle the egg-associated granulomatous pathway was through more frequent or earlier treatment rather than targeting the eggs directly. Treatment with anti-inflammatories in combination with new anti-schistosomal drugs may be suitable for dealing with morbidity.^28^

### Fast or Slow Kill?

There is anecdotal evidence to suggest that the administration of PZQ to an individual that is heavily infected may induce adverse effects, including severe abdominal pain. This is presumably due to a large number of paralysed worms being swept into the liver (where they are eventually killed) and releasing immune-stimulating antigens.^29^ More substantial evidence needs to be obtained around this, including whether this is observed with all schistosome species and whether the effect is related to infection intensity.

If this evidence is validated, then it would be a reasonable conclusion that a slower-acting compound would be preferable to mitigate the potential issue of adverse effects. However, developing a single-dose treatment which is slow-acting could be challenging. A compound of this type would need to either have a long half-life or be able to induce a rapid, irreversible effect on the parasite in which the parasites take a while to die and be cleared from the body. Nevertheless, if a long-acting compound with a slow rate of kill were to be developed, it could be efficacious and potentially provide a further advantage of allowing for a more sustained, trickle-release of dead worm antigens. This may lead to a greater chance of inducing a naturally evoked protective immune response upon reinfection. A key challenge in identifying fast- or slow-acting anti-schistosomals is the quantification of the rate of kill. Development of washout experiments will also be invaluable in terms of further enriching our understanding of anti-schistosomal mechanisms and modelling pharmacokinetic/pharmacodynamic relationships.

## Screening Cascade

### Critical Assays to Support the Drug Discovery Pathway

Drug discovery for small molecule inhibitors of *Schistosoma spp.* is hampered by low assay throughput, due to the limited supply of adult and juvenile worms. Methods for determining the effect of compounds on the worms can also be time-consuming. Current *ex vivo* screening platforms used by researchers at Aberystwyth University and the University of California, San Diego take different, yet complementary approaches to small molecule screening.

The Caffrey group (UC San Diego) employs an *S. mansoni* adult worm screen that concomitantly measures worm motility and morphological changes. Although this assay platform supports screening against the most relevant stage of the schistosome lifecycle, it has limited throughput because adult worms must be harvested from infected rodents. An additional complexity of adult worm assays is that they rely on microscopic examination to determine changes to worm motility and phenotype upon compound treatment. As an approach to increasing assay throughput, adult worm screens are conducted using mixtures of compounds followed by hit deconvolution to identify the active compound(s). This approach of combining observational analysis and measuring worm motility via WormAssay^30^ has proven consistent in identifying hits and generating useful structure–activity relationships.^31,32^ The importance of testing compounds against endemic schistosome strains was stressed. There is currently a large collection of clinical isolates being collated by the Natural History Museum in London. In addition to this, a number of groups in Brazil possess a variety of parasite isolates, including those less sensitive to PZQ.^33^

The Hoffmann group (Aberystwyth University) use an alternative platform which enables higher throughput screening in 384-well format against *S. mansoni* schistosomula. Using an automated, high-content imaging platform which quantifies both schistosomula phenotype and motility, large collections of compounds can be rapidly progressed for anti-schistosomal triaging. This platform was originally developed by Quentin Bickle and colleagues,^34^ but has been further developed in Aberystwyth to drive the identification and progression of new compounds for entering the drug discovery pipeline.^31^ A drawback to this higher throughput approach is that compounds targeting the schistosomula stage do not always show activity against adult worms.

The Hoffmann group are also investigating the use of approaches based on artificial intelligence and multi-modal image analysis, which should provide more information per assay and represent a more nuanced approach for identifying anti-schistosomals with diverse modes of action. Implementing these novel technologies as part of the screening cascade should provide more complex and robust assay information.

In addition to *ex vivo* screening platforms, *in vivo* models of infection are available. For example, compounds can be assessed in mouse models of *S. mansoni* infection to determine their effect on clearing juvenile and adult worms. The Hsieh group at George Washington University have also developed a mouse model of FGS which could help enhance understanding of the disease and reveal insights into the connection between FGS and HIV. To model FGS, a submucosal injection of eggs into the vagina is performed. From two weeks post-infection, granuloma formation occurs and increased voiding frequency is observed, thought to be due to pelvic pain. This model lacks cervical pathology as the egg deposition is artificial and does not lead to epithelial lesions. In addition, as there are no worms, it is not possible to test compounds in this model to monitor the effects of treatments. However, it has the potential to reveal information about the pathology of the disease which has so far been poorly understood for FGS.

## Human Challenge Model

Researchers at Leiden University Medical Centre have developed a human challenge model^35^ which could be pivotal for the clinical development of new anti-schistosomal drugs. It uses cercariae of a single sex to prevent egg formation, and infection is carried out by applying 10–30 cercariae directly to the skin. Initial adverse events are mild, with symptoms of acute schistosomiasis (night sweats, headache, fever) developing at around 4–5 weeks. Rescue treatment with 40 mg/kg PZQ was used for the male human-controlled infection leading to full cure in 10 out of 17 infected volunteers (59%). A second treatment of 60 mg/kg PZQ was needed to achieve cure. This model is now being transferred to Uganda for use in repeat infection studies and vaccine testing.

## Current Drug Discovery Activity

From a review of the literature, it appears that many drug discovery activities for schistosomiasis are focussed on screening and identifying early leads.^36−38^ To the best of our knowledge, the most advanced compound emanates from a project between the London School of Hygiene and Tropical Medicine (LSHTM) and Salvensis, and is now being developed by Merck KGaA, Darmstadt, Germany,.^39^ The criteria that Salvensis employed to progress their compound series to preclinical candidate selection were presented during the meeting. Significant increases were made to potency during hit-to-lead optimisation which also led to improvements in the cellular selectivity window. Pleasingly, these compounds were equipotent in juvenile and adult worms and showed activity against *S. mansoni*, *S. haematobium*, and *S. japonicum*. Efficacy was observed against both juvenile and adult worms in mouse *in vivo* efficacy models, and compounds displayed high oral bioavailability and long half-lives. The nominated preclinical candidate from this work has been onboarded by Merck KGaA, Darmstadt, Germany, and is currently undergoing preclinical safety studies along with active product ingredient (API) manufacturing, with the aim of progressing to Phase I trials.

**Figure 1:**
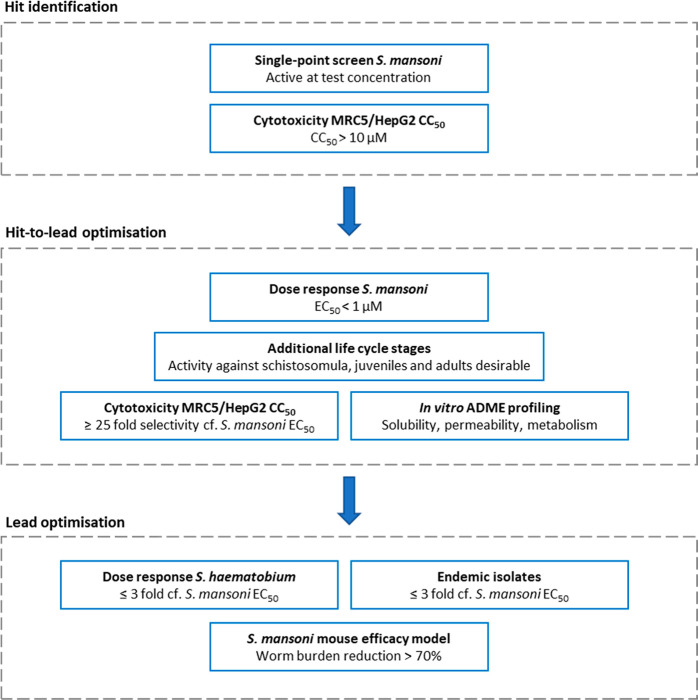
Summary of schistosomiasis-specific compound progression criteria employed by drug discovery projects at Calibr–UC San Diego, Salvensis, Cardiff University, Aberystwyth University, and University of Dundee.

Projects at a much earlier phase are being conducted by the Universities of Dundee, Aberystwyth, and Cardiff. Aberystwyth/Cardiff Universities have reported a compound series that demonstrates potent activity against schistosomes (schistosomula, juveniles, and adults; *S. mansoni*, *S. japonicum*, and *S. haematobium*),^31^ but requires optimisation of PK properties. A team from Calibr at Scripps Research are collaborating with the Caffrey group at UC San Diego and researchers at the Universities of Franca and Campinas in Brazil to screen the Re-FRAME library,^40^ a drug-repurposing collection of around 12,000 compounds. There may be other drug discovery activities being carried out by groups that were not present at this meeting. A summary of screening cascades showing schistosomiasis-specific compound progression criteria used by Calibr-UC San Diego, Salvensis, Cardiff University, Aberystwyth University, and University of Dundee are shown in Figure 1.

Our analysis of currently published anti-schistosomal compounds indicates that these tend to be lipophilic with low polar surface areas. It is uncertain whether these properties are a prerequisite for anti-schistosomal activity (due to the parasite’s heptalaminate surface membranes)^41^ or if they are a consequence of the compound collections that have been screened. High lipophilicity and low polar surface area could lead to developability issues due to the association of these properties with increased cytotoxicity, higher rate of metabolism, and potential for off-target effects.

## Target-Based Drug Discovery

Given the challenges of high throughput screening against the whole organism, target-based screening is an attractive strategy for schistosomiasis drug discovery. However, it is important that potential drug targets should be validated, and work in the Collins and Hoffmann laboratories is addressing this. The Collins laboratory at UT Southwestern Medical Center has successfully carried out large-scale RNAi experiments^42^ to examine the function and essentiality of genes to the survival of adult worms. Further work is ongoing to prioritise targets based on their druggability.

A successful way to validate targets in other organisms has been target deconvolution of phenotypically active compounds.^43,44^ This is a technique that is just being developed in schistosomes. Many of the genetic tools (e.g., genome editing) that have been successfully used in other pathogens are not easily translatable to schistosomes due to their complex life cycle. However, tools such as thermal proteome profiling and pull-down experiments using chemical proteomics are feasible and are currently being developed.

## Outstanding Questions

Despite the progress made in developing screening platforms to identify new anti-schistosomals, there are many outstanding questions that must be addressed to expand our understanding of this disease area and allow us to design suitable new treatments.

There is a lack of evidence available on the likelihood of resistance generation to PZQ in the field. If this were to occur to any substantial extent, it would have a devastating effect on prevention and treatment of schistosomiasis, emphasising the need for alternative and more targeted treatments.

Due to the different locations of parasites within the body, which vary with *Schistosoma* species and individual life cycle stages, it is important to establish desirable compounds properties that will be required to achieve elimination. However, the question of whether a compound should have a fast or slow rate of kill remains to be addressed.

It is not known how readily compounds can penetrate into schistosomes and whether compound efflux occurs, as is seen in Gram-negative bacteria. If compound permeability/efflux is an issue, what is the effect of physicochemical properties on compound exposure in the parasites, and how does this differ between different life cycle stages?

Given the challenges associated with testing compounds in whole organism assays, it would be helpful to develop target-based assays. Work is ongoing to identify suitable targets for small molecule drug discovery that are essential for parasite survival and relevant across all human life cycle stages.

In general, urogenital schistosomiasis (leading to FGS and MGS) is poorly understood. A greater knowledge of the pathology of these diseases is critical in understanding how to treat them, particularly the morbidity due to granulomas and fibrosis. Could anti-inflammatories have a potential role in managing the morbidity of FGS and MGS? In addition, the link between FGS/MGS and the onset and progression of cervical and prostate cancer or HIV is not well understood and further research is needed in this area.^45^ While our discussion sessions focussed on urogenital schistosomiasis, there are other forms such as pulmonary and neurological schistosomiasis which are also poorly characterised.

## Conclusions

In over 40 years of clinical use, PZQ has been the main operational approach to reducing the prevalence of schistosomiasis, but to achieve elimination (and eradication) an integrated approach including other interventions is necessary. Additional drug(s) will be needed, especially if immunoprophylactic vaccines are not developed and widespread resistance to PZQ emerges. It is necessary to carry out surveillance to monitor whether resistance to PZQ arises, particularly as new schistosome strains emerge. Recent data suggests that there needs to be a review of diagnostics for schistosomiasis and that current methods have under-reported cases of the disease. New drugs, at the very least, will need to be active against adults and juveniles, with anti-schistosomula activity an additional bonus. There is still much to be learned about treatment of schistosomiasis, and the road to developing new anti-schistosomal drugs remains a challenge, not least by the lack of an international product development partnership for a disease that affects hundreds of millions of people worldwide. Worryingly, there is no clear sign that the resources to accelerate schistosomiasis drug discovery are forthcoming.

Encouragingly, however, Merck KGaA, Darmstadt, Germany, and partners have demonstrated a pathway to the clinic for pediatric PZQ, which is important for treating infants and small children. With the LSHTM/Salvensis drug candidate, there is now a pathway for future clinical development of a new anti-schistosomal. These welcome changes and the increased collaborative engagement of the schistosome drug development community harnessed, for example, by this meeting in London should pave the way for a pipeline of small molecules in the future. The authors welcome feedback and engagement from anyone reading this Perspective.

## Supporting Information Available

Meeting Program of the Schistosomiasis Drug Discovery Workshop (PDF)
