# Supplementary material for: Perspective on Schistosomiasis Drug Discovery: Highlights from a Schistosomiasis Drug Discovery Workshop at Wellcome Collection, London, September 2022
Source: ACS Infect Dis. 2023 Apr 21;9(5):1046–55. doi: 10.1021/acsinfecdis.3c00081 (PMC10186373; doi:10.1021/acsinfecdis.3c00081)
Supplement: Supplementary file 1 — id3c00081_si_001.pdf [file id3c00081_si_001.pdf]

Schistosomiasis Drug Discovery Workshop

Wellcome Collection, London

22nd – 23rd September 2022

Meeting Program

Thursday 22nd September

**09.20 – 10.00**	**Arrival**		
09.20	Arrival and welcome		
09.40	Ian Gilbert	Opening remarks	
**10.00 – 11.20**	**Session 1**	**Current progress with new treatments – key compounds and human trials**	*Chair: Nicola Caldwell*
10.00	Rana Afshar	Arpraziquantel development program update	
10.20	Thomas Spangenberg	Update on clinical progress of Salvensis compound	
10.40	Nathan Lo	Modelling of novel drugs against Schistosoma	
11.00	Panel discussion with all session 1 speakers		
**11.20**	**Break**		
**11.40 – 13.00**	**Session 2**	**Urogenital schistosomiasis – key requirements for new treatments**	*Chair: Amaya Bustinduy*
11.40	Mireille Gomes	FGS	
12.00	Daniela Fusco	FGS and key requirements for new treatments	
12.20	Michael Hsieh	MGS and in vivo models for FGS	
12.40	Panel discussion with all session 2 speakers		
**13.00 – 14.00**	**Lunch**		
**14.00 – 14.50**	**Session 3**	**Target product profiles for FGS and MGS**	*Chair: Sabine Specht + Daniela Fusco*
14.00	Discussion session		
**14.50 – 16.50**	**Session 4**	**Drug discovery pathway and critical assays**	*Chair: Kevin Read*
14.50	Karl Hoffmann	Aberystwyth University screening platform	
15.10	Conor Caffrey	UC San Diego screening platform	
**15.30**	**Break**		
15.50	Jim Collins	RNAi screening	
16.10	Meta Roestenberg	Human challenge model	
16.30	Panel discussion with all session 4 speakers		
**16.50 – 17.00**	**Day 1 finish**		

Schistosomiasis Drug Discovery Workshop

Wellcome Collection, London

22nd – 23rd September 2022

Meeting Program

Friday 23rd September

**09.00 – 10.00**	**Session 5**	**Defining a target product profile for schistosomiasis**	*Chair: Beatriz Baragaña*
09.00	Justin Nono Komguep	Key requirements for new treatments in endemic areas	
09.20	Amadou Garba Djirmay	WHO perspective on new drugs for schistosomiasis	
09.40	Panel discussion with all session 5 speakers		
**10.00 – 10.50**	**Session 6**	**Target product profiles for schistosomiasis**	*Chair: Sabine Specht + Ian Gilbert*
10.00	Discussion session		
**10.50 – 11.10**	**Break**		
**11.10 – 12.50**	**Session 7**	**Drug discovery and compound progression criteria**	*Chair: Karl Hoffmann*
11.10	Gilda Padalino	Schistosomiasis drug discovery at Cardiff University	
11.30	Nicola Caldwell	Schistosomiasis drug discovery at Dundee University	
11.50	Case McNamara	Calibr workstream and screening	
12.10	Mark Gardner	Progression criteria in the discovery of the Salvensis series	
12.30	Panel discussion with all session 5 speakers		
**12.50 – 13.00**	**Closing remarks**		
12.50	Ian Gilbert	Closing remarks	
**13.00 – 14.00**	**Lunch**		
